# Supplementary material for: Identification of photoperiod-induced specific miRNAs in the adrenal glands of Sunite sheep (Ovis aries)
Source: Front Vet Sci. 2022 Jul 22;9:888207. doi: 10.3389/fvets.2022.888207 (PMC9354845; doi:10.3389/fvets.2022.888207)
Supplement: Supplementary file 1 [file Data_Sheet_1.ZIP › Supplementary file/miRDeep2 file.zip!/miRDeep2 file/Know_Structure/SP42A1/oar-mir-376e.pdf]

miRBase precursor	: oar-mir-376e
Total read count	: 944
oar-miR-376e-5p read count	: 164
oar-miR-376e-3p read count	: 616
oar-miR-376b-5p read count	: 164
remaining reads	: 0

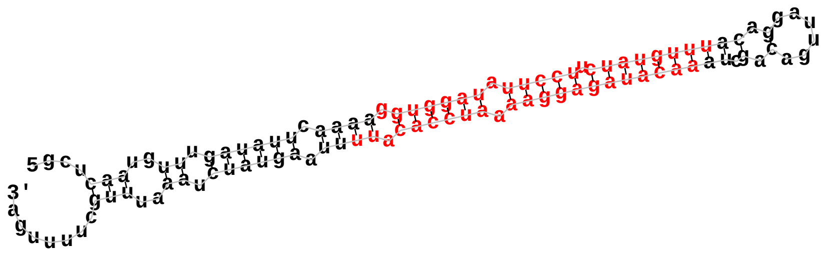

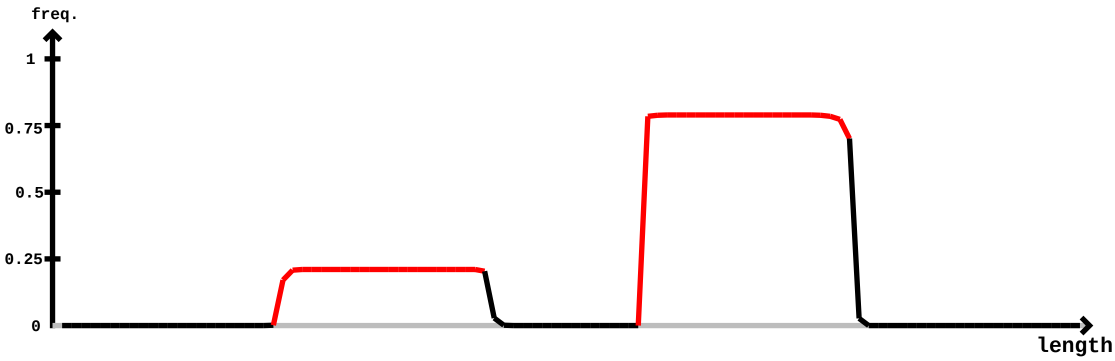


```
                                                                oar-miR-376e-3p
                        oar-miR-376b-5p
                        oar-miR-376e-5p
5'- gcucaauguuugauauucaaaagguggauauuccuucuauguuuacaggauugacagcuaaacauagaggaaaauccacauuuuaaguaucuaaaauuugcuuuga  -3'   exp
    ((.....((((((((((.((((.((((((.(((((.((((((((((.(......).).)))))))))))))).)))))).)))).)))))).))))..))......   reads   mm   sample
    .....................agguggauauuccuucuauguuu..............................................................   1       0    seq
    ......................gguggauauuccuucuauguu...............................................................   5       0    seq
    ......................gguggauauuccuucuauguuu..............................................................   112     0    seq
    ......................gguggauauuccuucuauguuua.............................................................   14      0    seq
    ......................gguggauauuccuucuauguuuac............................................................   1       0    seq
    .......................guggauauuccuucuauguuu..............................................................   22      0    seq
    .......................guggauauuccuucuauguuua.............................................................   7       0    seq
    ........................uggauauuccuucuauguuu..............................................................   2       0    seq
    ............................................................aacauagaggaaaaucca............................   1       0    seq
    ............................................................aacauagaggaaaauccac...........................   3       0    seq
    ............................................................aacauagaggaaaauccaca..........................   9       0    seq
    ............................................................aacauagaggaaaauccacau.........................   56      0    seq
    ............................................................aacauagaggaaaauccacauu........................   523     0    seq
    ............................................................aacauagaggaaaauccacauuu.......................   20      0    seq
    .............................................................acauagaggaaaauccacauu........................   3       0    seq
    ..............................................................cauagaggaaaauccacauuu.......................   1       0    seq
```
